# Chylothorax: A Late Complication of Disseminated Mycobacterium avium Complex (MAC) Infection

**DOI:** 10.7759/cureus.40347

**Published:** 2023-06-13

**Authors:** Abhishrut Jog, Patrik Schmidt, Priscilla L Hallal, Richard Novitch

**Affiliations:** 1 Pulmonary Medicine, BronxCare Health System, Bronx, USA; 2 Internal Medicine, BronxCare Health System, Bronx, USA

**Keywords:** immune reconstitution inflammatory syndrome, mycobacterium avium complex, hiv, chylous ascites, chylothorax

## Abstract

Chylothorax is a rare cause of pleural effusion and occurs due to leakage of chyle into the pleural space. In most cases, it results from trauma, with malignancy accounting for most of the non-traumatic causes. Chylothorax resulting from immune reconstitution inflammatory syndrome (IRIS), during treatment of *Mycobacterium avium* complex (MAC) infection, is an extremely infrequent cause of chylothorax, with only a handful of cases reported in the literature.

## Introduction

Chylothorax is a rare cause of pleural effusion caused by the leakage of chyle from the thoracic lymphatic circulation into the pleural space. In most cases, it results from a form of trauma [1]. Recognition of chylothorax and chylous ascites in immunocompromised patients is crucial, as nutritional, and immunological deficiencies resulting from these conditions may lead to worse clinical outcomes. Chylothorax resulting from IRIS, during treatment of *Mycobacterium avium* complex (MAC) infection, is an extremely infrequent cause of chylothorax [2], with only a handful of cases reported in the literature. Even rarer is the presence of concomitant chylous ascites. We present one such case of chylothorax and chylous ascites in a patient with disseminated MAC. 

## Case presentation

A 48-year-old male with a past history of human immunodeficiency virus (HIV) infection and disseminated MAC infection (on treatment), was evaluated for a newly developed right-sided pleural effusion. He had a history of recurrent hospital admissions for ascites that had occurred over a period of 6 months prior to this presentation.

He had been diagnosed with disseminated MAC around 2 years prior, on a bone marrow biopsy, and had been on treatment with ethambutol, rifabutin, and azithromycin since then. His CD4 count at the time of initiation of treatment for MAC had been 20 cells/mm3, and his viral load had been 9980 copies/mL. Over the next 18 months, his CD4 count steadily increased to 344 cells/mm3 and viral load became undetectable.

It was around this time when his CD4 count had improved to 344 cells/mm3 that he started presenting with recurrent ascites. Paracenteses on three occasions showed milky ascetic fluid with triglyceride levels of 238, 172, and 391 mg/dL, suggesting chylous ascites. 

**Figure 1:**
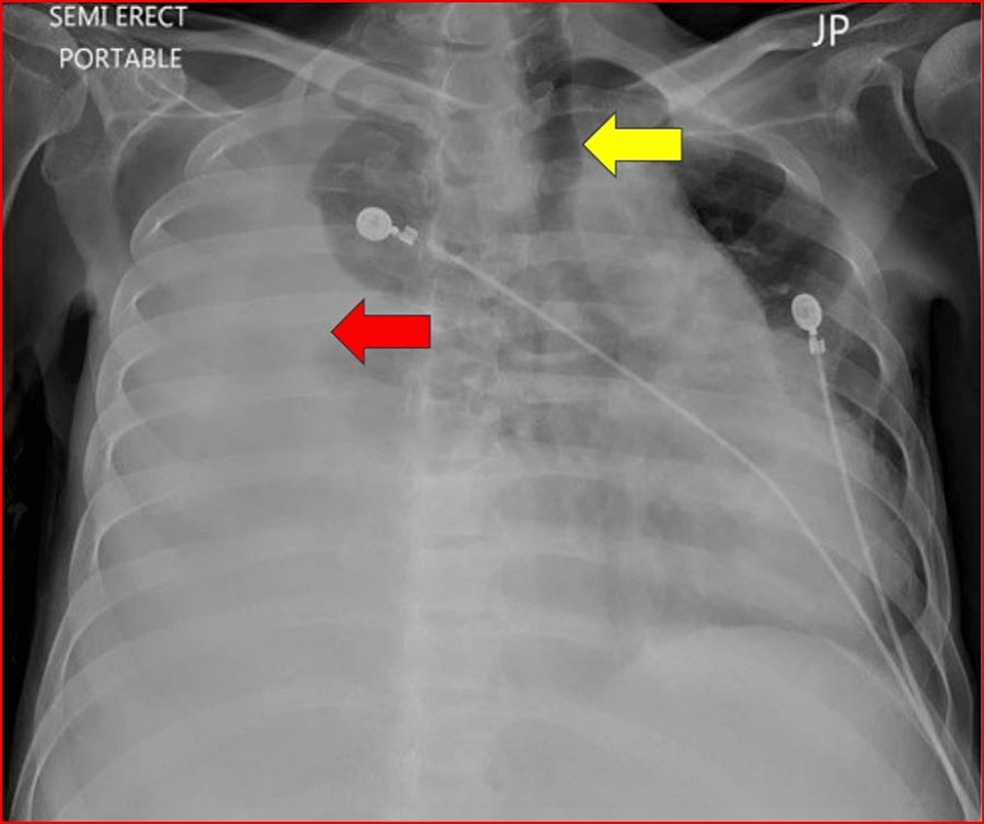
Chest X-ray with right-sided homogenous opacification (red arrow) and mediastinal shift to the opposite side (yellow arrow) suggesting an effusion.

Subsequently, he presented with complaints of worsening shortness of breath, persistent cough, and worsening abdominal distention. On physical examination, he had reduced breath sounds on the right with dullness to percussion, and shifting dullness over the abdomen. His initial chest X-ray revealed a large right homogenous opacification with a meniscus sign and with the mediastinal shift (Figure 1).

**Figure 2:**
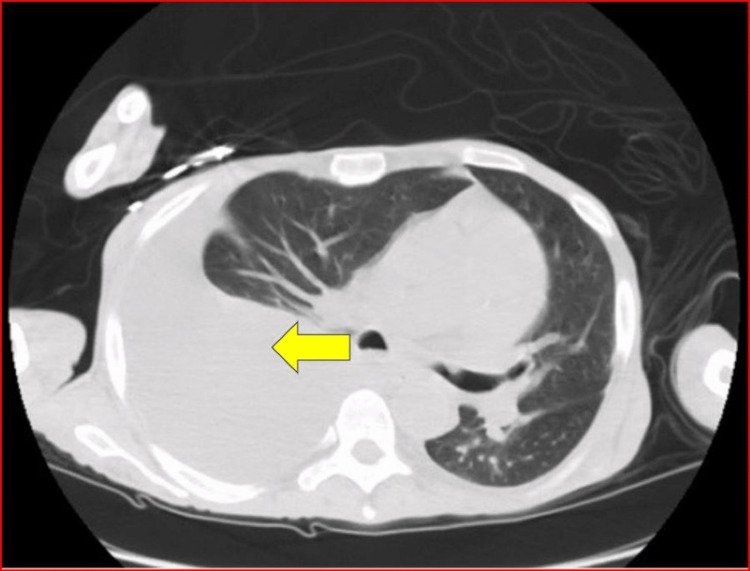
CT chest with right pleural effusion (yellow arrow).

CT chest corroborated the findings (Figure 2). 

**Figure 3:**
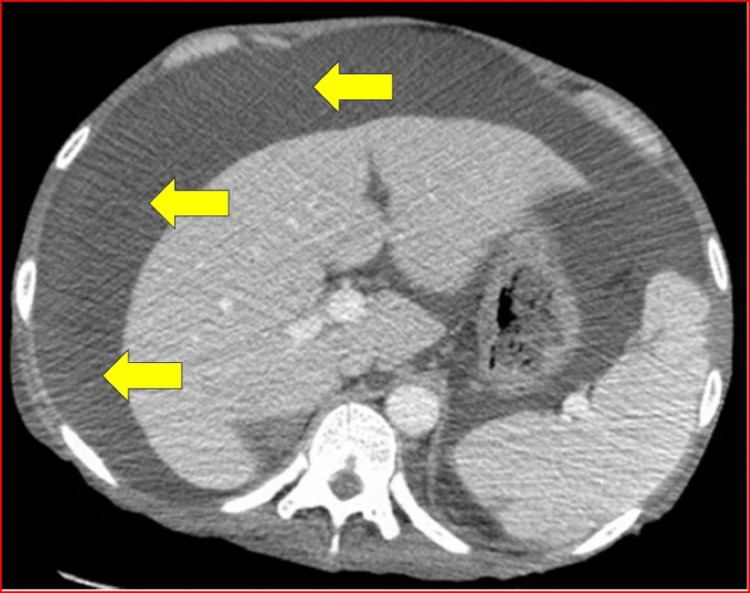
CT abdomen with ascites (yellow arrows).

A CT scan of the abdomen again revealed large ascites with significant adenopathy (Figure 3).

He underwent thoracentesis. The fluid was grossly milky in appearance (Table 1). The pleural fluid studies revealed a triglyceride level of 405 mg/dL and a cholesterol level of 28 mg/dL, confirming a diagnosis of chylothorax. He underwent a therapeutic paracentesis, again revealing chylous fluid.

**Table 1:** Thoracentesis findings. RBC, red blood cell; WBC, white blood cell; ADA, adenosine deaminase; LDH, lactate dehydrogenase

Characteristic	Result	Reference values
Appearance	Cloudy	Straw colored
pH	8.00	7.60-7.64
WBC count (cells/mm^3^)	26	< 1000
RBC count (mil cells/mm^3^)	198	Variable
Neutrophil count (%)	8	<1
Lymphocyte count (%)	92	23
Albumin	0.3	Variable
Amylase (unit/L)	47	30-110
Glucose (mg/dL)	126	Plasma level
LDH (units/L)	46	<50% Plasma level
Protein (g/dL)	1	1-2
Triglyceride (mg/dL)	405	<110
Cholesterol (mEq/L)	28	Variable
ADA (U/L)	4.4	<9.2

There were no traditional risk factors for chylous ascites or chylous pleural effusions such as trauma, surgery, or lymphoma. The CT scan finding of significant abdominal lymphadenopathy and the increasing levels of CD4 cells and undetectable viral load provided evidence of IRIS from MAC as a cause of the chylous effusions. The patient was continued on HAART and antimycobacterial therapy. Dietary supplements were initiated. The pleural effusion and the ascites subsided over the next few weeks. 

## Discussion

Chylothorax is a condition defined as the presence of chyle, a milky fluid rich in fat and lymph, within the pleural space [1]. Chylothorax (or chylous ascites) are diagnosed by the presence of triglyceride levels >110 mg/dL, low cholesterol levels, and the presence of chylomicrons in the ascitic or pleural fluid [2].

Etiologies are typically divided into two primary mechanisms -- nontraumatic and traumatic [1, 3]. Nontraumatic causes can be malignant and nonmalignant, while traumatic causes are often attributed to surgical procedures. As chyle contains triglycerides, immune cells, electrolytes, proteins, and nutrients in the form of vitamins and minerals [4], untreated, chylothorax can predispose patients to serious infections and malnutrition. Accumulation of fluid in the pleural space can lead to respiratory complications such as increased work of breathing, respiratory compromise, prolonged ventilation time, and increased mortality [1].

Mycobacterial infections have been documented as a cause of chylous ascites in patients with AIDS, and the presence of chylous ascites has been observed as a late complication of intra-abdominal MAC immune reconstitution syndrome [5-7]. Our patient had rising CD4 counts from 38 to 344, with undetectable HIV viral levels, persistent lymphadenopathy, and worsening ascites. These changes suggest the interval development of IRIS.

The development of chylous ascites and pleural effusion is linked with the anatomical location of the cisterna chyli. The cisterna chyli is anatomically located in between the abdominal aorta and the first two lumbar vertebrae. Granulomatous lymphadenitis from the mycobacterial infection is thought to result in lymphatic obstruction at the base of the mesentery or cisterna chyli, leading to chylous ascites [6]. The pleural effusion occurred as a consequence of these ascites.

In a case series by Phillips et al., of non-tubercular mycobacteria-related IRIS, 62% of patients with the intra-abdominal disease were preceded by disseminated MAC infection, as seen in our case. In this series, all cases of intra-abdominal MAC had lymphadenopathy, as in our case. Although the average time to onset of IRIS from initiation of HAART was 3 weeks in this series, it may be delayed by months to years. Late-onset IRIS occurred after 2 years in this case series [5]. In a case series of 20 patients with IRIS due to MAC infection studies by Riddell et al., 35% of patients developed IRIS less than 60 days after initiation of HAART, 50% from 60 days to one year, and 15% after more than a year [8]. In our case, the IRIS occurred almost 18 months after treatment initiation for MAC.

Subsequent to the recurrent chylous ascites and chylothorax, the patient’s CD4 counts dropped transiently. We believe that this might have occurred due to the loss of lymphocytes from the blood into the lymphocyte-rich chylous fluid.

Management of chylothorax includes placement of a pleural drain to remove the chyle to alleviate respiratory distress, along with close monitoring of serum electrolytes, protein, and lymphocyte count (as these are lost through the chyle). The same is true for chylous ascites. Dietary modifications, specifically a low fat/high protein diet with supplemented medium chain triglycerides may further help decrease the flow of chyle, thus reducing the rate of chyle re-accumulation [9]. Somatostatin and octreotide are also useful as adjuvant therapy as they ultimately reduce the amount of chyle production and flow rate, and can help avoid the need for surgical repair [10-13]. In patients with high-output chyle leaks, thoracic duct ligation or embolization may be warranted within hours, as mortality in these patients is especially high. In patients who have failed dietary, symptomatic, and even surgical treatment options, midodrine and other alpha-1 adrenoreceptor agonists have been shown in case reports to be effective in further reducing chylous drainage [14-15]. To reduce ascites, the placement of an intrahepatic portosystemic shunt used to decrease portal pressures in cirrhotic patients can also be helpful [16].

The most important component in the management of chylothorax and chylous ascites is to treat the underlying etiology of the disease. Recognizing and appropriately managing chylothorax and chylous ascites in immunocompromised patients can help reduce nutritional and immunological losses from chylous drainage, which may lead to improved clinical outcomes.

## Conclusions

Chylothorax and chylous ascites can occur as a part of IRIS during the treatment of disseminated MAC infection. The complication might have a very delayed onset. It is important to recognize this late presentation of IRIS in the setting of MAC treatment.
